# Epidemiology of quid usage and its possible association with the occurrence of oral mucosal lesions

**DOI:** 10.3389/froh.2024.1450729

**Published:** 2024-11-27

**Authors:** Sultan A. Almalki, Inderjit M. Gowdar, Manoj Vengal, Sugandha Arya

**Affiliations:** ^1^ Department of Preventive Dental Sciences, College of Dentistry, Prince Sattam Bin Abdulaziz University, Alkharj, Saudi Arabia; ^2^ Department of Oral Medicine and Radiology, KMCT Dental College, Kozhikode, India; ^3^ Department of Oral Medicine and Radiology, Vyas Dental College and Hospital, Jodhpur, India

**Keywords:** quid usage, oral mucosal lesions, betel quid, oral submucous fibrosis, leukoplakia, tobacco pouch keratosis, western Rajasthan, public health

## Abstract

**Background:**

Chewing tobacco consumption has become a major public health issue in India. The incidence of oral cancer is increasing in India, especially among young adults.

**Aim:**

The objective of this study was to ascertain the prevalence of chewing tobacco consumption and its association with the occurrence of oral mucosal lesions.

**Methods:**

A cross-sectional survey was conducted among 1,209 patients who reported to the Department of Oral Medicine and Radiology at Vyas Dental College, Jodhpur. A structured questionnaire was used to collect information on patients’ personal information such as demographic details, tobacco use, and other adverse habits. A single calibrated investigator performed a clinical examination of lesions on the oral mucosa, and the diagnosis was further validated.

**Results:**

The majority of the participants were men (81.8%) in the age range of 26–35 years. A habit of chewing a combination of tobacco and areca nut (48.2%) was more prevalent. More than 36% of the participants consumed tobacco in a processed form (90%), at least four times a day. It was observed was 25% of the participants were suffering from tobacco pouch keratosis.

**Conclusion:**

Chewing tobacco was significantly associated with the development of oral mucosal lesions, particularly among men and those belonging to lower socio-economic groups. Along with targeted public health interventions on tobacco cessation, it is essential to change the cultural norms associated with the habit of chewing tobacco and implement strict chewing tobacco control laws in the community and workplaces.

## Introduction

The use of quid—a term encompassing a variety of chewing substances such as betel quid, tobacco, and areca nut—has been a practice embedded in various cultures for centuries. Particularly prevalent in South and Southeast Asia, quid usage has permeated global communities due to migration and cultural dissemination. While this traditional practice carries significant cultural and social weight, it has also been increasingly scrutinized for its potential health impacts, particularly its association with oral mucosal lesions (OMLs). Oral mucosal lesions, which include conditions such as leukoplakia (LP), erythroplakia, and oral submucous fibrosis (OSMF), are not merely benign afflictions; they often carry a premalignant potential, raising concerns about oral cancer (1–3).

The epidemiology of quid usage reveals a complex interplay of socio-economic, cultural, and behavioral factors that influence its prevalence and patterns. Understanding these dynamics is crucial for public health interventions aiming to mitigate the risks associated with quid usage. This introduction delves into the epidemiological landscape of quid usage, examining its prevalence, socio-demographic determinants, and the biological mechanisms underpinning its association with OMLs. Recent studies will be referenced to provide a comprehensive overview, highlighting the latest findings and gaps in current knowledge.

## Epidemiological landscape of quid usage

Quid usage, particularly betel quid, is widespread in many parts of the world. In countries such as India, Pakistan, Bangladesh, Sri Lanka, and Taiwan, the practice is not only common but culturally significant. The Global Adult Tobacco Survey (GATS) reports that over 600 million people globally use betel quid, with the highest prevalence observed in Southeast Asia (4). In India alone, approximately 20%–40% of the population is estimated to be regular users of betel quid (5). The reasons for its usage are multifaceted, including cultural traditions, stress relief, increased alertness, and socio-economic factors.

## Socio-demographic determinants

The demographic profile of quid users varies, but certain patterns are evident. Gender differences are prominent, with men being more likely to use quid than women, although the gap is narrowing in some regions (6). Age is another critical factor; usage often begins in adolescence and peaks in middle age. Socio-economic status (SES) also plays a significant role. Lower socio-economic groups have higher rates of quid usage, often due to the affordability and accessibility of quid compared to other stimulants like cigarettes or alcohol (7, 8).

## Biological mechanisms and oral mucosal lesions

The pathogenesis of OMLs in quid users involves several biological mechanisms. The primary components of quid—areca nut, betel leaf, and tobacco—contain a multitude of carcinogenic substances. Areca nut, for instance, contains alkaloids such as arecoline, which are known to induce fibroblast proliferation and collagen synthesis, leading to conditions such as OSMF (9).

Tobacco, whether in smoke-less or smoked form, contributes additional carcinogens such as nitrosamines, which exacerbate the risk of malignancies (10).

Oral mucosal lesions manifest in various forms among quid users. Leukoplakia, characterized by white patches on the oral mucosa, is one of the most common lesions. Its prevalence among betel quid users ranges from 5% to 30%, depending on the intensity and duration of usage (11). Although less common, erythroplakia has a higher malignant potential, with studies indicating a transformation rate of up to 50% (12). Oral submucous fibrosis, a chronic and potentially malignant condition, is predominantly seen in South Asian populations, with an estimated prevalence of 2%–8% among quid users (13, 14).

Recent epidemiological studies have shed light on the intricate relationship between quid usage and the occurrence of OMLs. A cohort study conducted in Taiwan followed 10,000 quid users over a decade, finding a significant increase in the incidence of oral cancer among users compared to non-users (15). Similarly, a study in India involving 5,000 participants revealed that the risk of developing leukoplakia was 10 times higher in quid users, with a dose-response relationship evident (16).

In addition to observational studies, molecular research has identified specific biomarkers associated with OMLs in quid users. For example, elevated levels of p53 mutations have been detected in the oral mucosa of chronic betel quid chewers, suggesting a potential pathway for malignant transformation (17).

Moreover, epigenetic modifications, such as DNA methylation changes, have been linked to the development of OSMF, highlighting the complex interplay of genetic and environmental factors in disease progression (18). The public health implications of these findings are profound. Given the high prevalence of quid usage and its strong association with OMLs and oral cancer, targeted interventions are necessary. Public health campaigns focusing on the risks associated with quid usage, combined with efforts to provide cessation support, are critical. Moreover, regulatory measures to control the sale and marketing of quid components, particularly to vulnerable populations like adolescents, could significantly reduce usage rates (19, 20). The objectives of present were (a) to determine the prevalence of chewing tobacco usage among the population of western Rajasthan, (b) to investigate the prevalence of oral mucosal lesions among the population of western Rajasthan, and (c) to find the association between oral mucosal lesions and chewing tobacco habit among the population of western Rajasthan.

## Materials and methods

### Study design

A descriptive cross-sectional survey was conducted on 1,209 patients who reported to the Department of Oral Medicine and Radiology at Vyas Dental College, Jodhpur.

### Ethical consent

The study received approval from the Institutional Review Board (IRB) of Vyas Dental College, Jodhpur. Written informed consent was obtained from all participants after they were thoroughly explained about the purpose of the study in simple and understandable language. This ensured that the participants were fully aware of the aim of the study and also their rights.

### Sample size estimation and sampling technique

The sample size for this study was calculated based on the prevalence of quid usage among North Indians, which was estimated at a rate of 52%. A marginal error of 0.05 was set, leading to a minimum required sample size of 1,209 participants. Over a period of one year, 6,400 subjects presenting to the Department of Oral Medicine and Radiology were screened. From this cohort, 1,209 quid users—who consumed processed, unprocessed, or both forms of tobacco and areca nut—were selected using a convenient sampling technique. Those subjects who had discontinued the quid habit for two years or more, were known to have other deleterious habits such as smoking, alcohol consumption, drug addiction, or consumption of other drugs, had amalgam or composite restoration, metallic crowns, and a history of graft placement, or were undergoing treatment for any oral mucosal lesions were excluded.

### Data collection

For all the 1,209 participants, case histories were taken in the local language, ensuring an accurate recording of chief complaints, medical histories, and sociodemographic factors. The socioeconomic status of the participants was assessed using the Kuppuswamy scale. Detailed information about their chewing habits—including the type and form of quid used—was recorded on a case sheet developed using a modified version of the WHO Oral Health Assessment Form. The participants were categorized into three main groups based on the type and contents of the quid and also subdivided into three groups according to the form of quid used (as presented in Tables 1 and 2). In addition, the frequency of quid usage per day, the duration of the chewing habit (in years), and the time (in minutes) that the quid was kept in the mouth each day were documented.

*Classification of oral mucosal lesions* - Quid-induced oral mucosal lesions were given numerical codes in the case sheet, which are as follows: Homogeneous leukoplakia (0), Non-homogeneous leukoplakia (1), Erythroplakia (2), Betel chewer’s mucosa (3), Quid-induced lichenoid reaction (4), Oral submucous fibrosis (5), Tobacco pouch keratosis (6), Carcinoma (7), and No lesion (8).

### Clinical examination

A clinical examination was performed using a sterile mouth mirror and sterile gauze packs under artificial lighting, following a standardized test protocol. All areas of the oral mucosa were assessed sequentially to identify quid-induced lesions. Clinical findings were evaluated using modified WHO criteria and confirmed by a qualified Oral Medicine and Radiology specialist. For participants with clinically diagnosed quid-induced lesions, incisional biopsies were recommended, and written informed consent was obtained from those who volunteered. Clinical photographs of the lesions were also taken, and habit counseling was provided, along with periodic recall checkups.

Calibration of the examiner: The clinical examination was conducted by a trained and calibrated examiner. The calibration process involved an oral diagnostician assigning 20 different quid-induced lesions for diagnosis, followed by a kappa analysis. Two weeks later, 10 of the same subjects were randomly selected and reassigned to the examiner to assess intra-examiner variability. The Kappa coefficient was found to be 0.9, indicating excellent agreement.

### Statistical analysis

Data were entered into Microsoft Excel and later exported to IBM SPSS Statistics for Windows (Version 22.0., Armonk, NY, USA) for a statistical analysis. The analysis was carried out by using a chi-square test, a bivariate analysis, and a multiple regression analysis. A multinomial regression analysis was carried out to understand the relationship between oral mucosal lesions (dependent variable) and independent variables such as quid form, quid type duration, and frequency of habits. Age, gender, and SES were considered as covariates. The significance level was fixed at p < 0.05.

## Results

Table 1 shows the demographic characteristics of the participants. The study involved participants primarily from western Rajasthan, with the most common age group being 26–35 years (28.8%), followed by 16–25 years (26.4%) and 36–45 years (22.1%). Men constituted a significant majority of the study population (81.8%), with a higher prevalence of tobacco use observed among men compared to women. SES was also assessed, revealing that the majority of participants belonged to the upper-lower (UL) and lower-middle (LM) socio-economic classes (36.1% and 28.9%, respectively). The combination of tobacco and areca nut was the most frequently used type, reported by 48.2% of participants. This combination is particularly concerning due to its strong association with the development of various OMLs. The processed form of tobacco was the most common, with 90.8% of participants reporting its use. Processed tobacco often contains additional chemicals and flavor enhancers, which can increase the risk of oral health issues. Furthermore, 36.9% of the participants reported chewing tobacco frequently, between 4 and 35 times a day. This high frequency is significantly associated with the prevalence of OMLs (*p* < 0.01). Most participants (45.7%) reported keeping the tobacco in their mouth for a moderate duration of 16–70 min. This moderate retention time is also significantly linked to the development of OMLs, with longer retention times (up to 1,440 min) correlating with more severe lesions. The history of tobacco use among the participants varied, with 49.3% reporting moderate-duration usage (24–108 months).

**Table 1:** Distribution of oral mucosal lesions according to socio-demographic details, quid type, quid form, and habit history.

Variable	Category	*N* (%)
Lesions	No lesion	260 (21.5)
	Leukoplakia	128 (10.5)
	Oral submucous fibrosis	254 (21)
	Betel chewer's mucosa	18 (1.4)
	Quid-induced lichenoid reaction	62 (5.1)
	Tobacco pouch keratosis	302 (24.9)
	Others	185 (15.3)
Age (years)	5–15	12 (1)
	16–25	328 (26.4)
	26–35	358 (28.8)
	36–45	274 (22.1)
	46–55	103 (8.3)
	56–65	67 (5.4)
	66–75	46 (3.7)
	>75	21 (1.7)
Gender	Women	220 (18.2)
	Men	989 (81.8)
Socio-economic class	Upper class	19 (1.5)
	Upper-middle class	46 (3.7)
	Lower-middle class	359 (28.9)
	Upper-lower class	448 (36.1)
	Lower class	337 (27.1)
Type of tobacco used	Tobacco	351 (29)
	Areca nut	275 (22.8)
	Both tobacco and areca nut	583 (48.2)
Tobacco form	Processed	1,097 (90.8)
	Unprocessed	48 (3.9)
	Both unprocessed and processed	64 (5.3)
Frequency of chewing per day (number of pouches)	Low (1)	364 (30.1)
	Medium (2–3)	399 (33)
	High (4–35)	446 (36.9)
Duration of chewing (min)	Low (1–15)	404 (33.4)
	Moderate (16–70)	553 (45.7)
	High (75–1,440)	252 (20.9)
Frequency of chewing in a month (number of pouches)	Less (1–18)	191 (15.9)
	Moderate (24–108)	597 (49.3)
	High (120–600)	421 (34.8)

Table 2 shows the bivariate analyses of various lesions according to socio-demographic details, quid type, quid form, and habit history. More oral mucosal lesions [LP, OSMF, quid-induced lichenoid reaction (QILR), and tobacco pouch keratosis (TPK)] were present in the 16–25, 26–35, and 36–45 year age groups compared to other age groups, and this difference was statistically significant (*p* < 0.01). Men were more affected than women by all the lesions (LP, OSMF, QILR, and TPK), and this difference was statistically significant (*p* < 0.01). Individuals from the UL and LM socio-economic classes were significantly more affected by various lesions, particularly LP, OSMF, QILR, and TPK.

**Table 2:** Bivariate analyses of oral mucosal lesions according to socio-demographic characteristics.

Variable	Category	No lesion (%)	LP (%)	OSMF (%)	BCM (%)	QILR (%)	TPK (%)	Others (%)	*p*-value
Age (years)	5–15	10 (0.8)	1 (0.1)	1 (0.1)	0	0	0	0	
	16–25	102 (8.4)	35 (2.9)	66 (5.5)	5 (0.4)	26 (2.2)	68 (7.4)	26 (2.2)	
	26–35	57 (4.7)	28 (2.3)	79 (6.5)	9 (0.7)	21 (1.7)	89 (7.4)	75 (6.2)	
	36–45	47 (3.9)	28 (2.3)	51 (4.2)	3 (0.2)	9 (0.7)	95 (7.9)	41 (3.4)	
	46–55	28 (2.3)	14 (1.2)	16 (1.3)	0	4 (0.3)	25 (2.1)	16 (1.3)	0.000*
	56–65	12 (1)	10 (0.8)	16 (1.3)	0	0	12 (1)	17 (1.4)	
	66–75	3 (0.2)	4 (0.3)	19 (1.6)	1 (0.1)	2 (0.2)	10 (0.8)	7 (0.6)	
	>75	1 (0.1)	8 (0.7)	6 (0.5)	0	0	3 (0.2)	3 (0.2)	
Gender	Women	48 (4)	15 (1.2)	72 (6)	3 (0.2)	12 (1)	22 (1.8)	48 (4)	0.000*
	Men	212 (17.5)	113 (9.3)	182 (15.1)	15 (1.2)	50 (4.1)	280 (23.2)	137 (11.3)	
SES	U	2 (0.2)	5 (0.4)	6 (0.5)	0	0	3 (0.2)	3 (0.2)	
	UM	5 (0.4)	4 (0.3)	18 (1.5)	0	2 (0.2)	10 (0.8)	7 (0.6)	
	LM	71 (5.9)	38 (3.1)	68 (5.6)	4 (0.3)	11 (0.9)	108 (8.9)	59 (4.9)	0.000*
	UL	82 (6.8)	42 (3.5)	95 (7.9)	8 (0.7)	22 (1.8)	113 (9.3)	86 (7.1)	
	L	100 (8.3)	39 (3.2)	67 (5.5)	6 (0.5)	27 (2.2)	68 (5.6)	30 (2.5)	

BCM, Buccal chewers mucosa.

* Statistically significant at *p* < 0.05.

** Statistically highly significant at *p* < 0.001.

Table 3 shows the bivariate analyses of various lesions according to quid type, quid form, and habit history The study found that the combination of tobacco and areca nut chewing was significantly more associated with the occurrence of OMLs, LP, OSMF, and TPK, compared to the use of tobacco alone (*p* < 0.001). The processed form of tobacco was frequently observed among participants with various OMLs, and this relationship was statistically significant (*p* < 0.001). High-frequency quid chewing (4–35 times per day) was significantly associated with an increased occurrence of OMLs (*p* < 0.001). The study demonstrated that longer durations of quid retention in the mouth (75–1,440 min) were associated with a higher occurrence of various OMLs (*p* < 0.01). Participants with a moderate duration of quid chewing habits (24–108 months) showed a statistically significant association with the occurrence of OMLs (*p* < 0.001).

**Table 3:** Bivariate analyses of various lesions according to tobacco type, form, and habit history.

Variable	Category	No lesion (%)	LP (%)	OSMF (%)	BCM (%)	QILR (%)	TPK (%)	Others (%)	*p*
Type	Tobacco	69 (5.7)	43 (3.6)	22 (1.8)	0	5 (0.4)	188 (15.6)	24 (2)	
	Areca nut	123 (10.2)	21 (1.7)	80 (6.6)	6 (0.5)	12 (1)	16 (1.3)	17 (1.4)	0.000*
	Both	68 (5.6)	64 (5.3)	152 (12.6)	12 (1)	45 (3.7)	98 (8.1)	144 (11.9)	
Form	Processed	238 (19.7)	113 (9.3)	235 (19.4	8 (0.7)	54 (4.5)	291 (24.1)	158 (13.1)	
	Unprocessed	9 (0.7)	4 (0.3)	11 (.9)	6 (0.5)	3 (0.2)	7 (0.6)	8 (0.7)	0.000*
	Both	13 (1.1)	11 (0.9)	8 (0.7)	4 (0.3)	5 (0.4	4 (0.3)	19 (1.6)	
Frequency of chewing tobacco in a day	Low	153 (12.7)	24 (2)	44 (3.6)	2 (0.2)	15 (1.2)	95 (7.9)	32 (2.6)	
	Medium	79 (6.5)	37 (3.1)	94 (7.8)	6 (0.5)	28 (2.3)	92 (7.6)	63 (5.2)	0.000*
	High	30 (2.4)	65 (5.4)	116 (9.6)	10 (0.8)	19 (1.6)	115 (9.5)	90 (7.4)	
Duration of quid in the mouth	Low ((1–15)	137 (11.3)	33 (2.7)	64 (5.3)	2 (0.2)	20 (1.7)	101 (8.4)	48 (4)	
	Moderate (16–70)	99 (8.2)	56 (4.6)	126 (10.4)	6 (0.5)	32 (2.6)	147 (12.2)	87 (7.2)	0.000
	High (75–1,440)	24 (2)	39 (3.2)	64 (5.3)	10 (0.8)	10 (0.8)	54 (4.5)	50 (4.1)	
Duration of chewing the quid (months)	Less (1–18)	73 (6)	14 (1.2)	33 (2.7)	4 (0.3)	14 (1.2)	39 (3.2)	14 (1.2)	0.000*
	Moderate (24–108)	133 (11)	66 (5.5)	120 (9.9)	9 (0.7)	36 (3)	153 (12.7)	80 (6.6)	
	High (120–600)	54 (4.5)	48 (4)	101 (8.4)	5 (0.4)	12 (1)	110 (9.1)	91 (7.5)	

BCM, Buccal chewers mucosa.

* Statistically significant at *p* < 0.05.

** Statistically highly significant at *p* < 0.001.

Table 4 contains the multinomial logistic regression showing the association between the dependent (lesions) and independent variables, the findings of which are as follows.

**Table 4:** Multinomial logistic regression showing association between dependent (lesions) and independent variables.

Lesions	Variables	Odds ratio (95% confidence interval)	*p*-value
Leukoplakia	Age	1.716 (2.204–1.336)**	0.000
	Sex	1.732 (3.358–0.893)	0.104
	Socio-economic status	1.492 (2.195–1.014)*	0.042
	Tobacco type	1.061 (1.405–0.801)	0.679
	Form	0.913 (1.437–0.580)	0.694
	Frequency of consumption	4.061 (5.923–2.784)**	0.000
	Duration of consumption per day	0.948 (1.409–0.637)	0.790
	Duration of consumption per month	1.377 (1.965–0.964)	0.078
Oral submucous fibrosis	Age	1.416 (1.776–1.129)**	0.003
	Sex	0.546 (0.868–0.344)**	0.010
	Socio-economic status	1.098 (1.537–0.785)	0.585
	Tobacco type	1.954 (2.511–1.520)**	0.000
	Form	0.601 (0.929–0.389)**	0.022
	Frequency of consumption	2.977 (4.098–2.163)**	0.000
	Duration of consumption per day	1.032 (1.442–0.739)	0.854
	Duration of consumption per month	1.546 (2.076–1.152)**	0.004
Quid-induced lichenoid reaction	Age	1.012 (1.943–0.527)	0.972
	Sex	0.645 (2.516–0.165)	0.527
	Socio-economic status	1.180 (3.077–0.453)	0.735
	Tobacco type	1.989 (4.628–0.855)	0.111
	Form	3.173 (5.945–1.693)**	0.000
	Frequency of consumption	3.173 (7.324–1.374)**	0.007
	Duration of consumption per day	1.789 (3.099–1.033)**	0.038
	Duration of consumption per month	0.769 (1.761–0.336)	0.534
Tobacco pouch keratosis	Age	1.124 (1.659–0.762)	0.556
	Sex	0.776 (1.622–0.371)	0.500
	Socio-economic status	1.280 (2.212–0.741)	0.376
	Tobacco type	2.672 (4.208–1.697)**	0.000
	Form	1.111 (1.907–0.648)	0.701
	Frequency of consumption	2.182 (3.550–1.342)**	0.002
	Duration of consumption per day	0.890 (1.498–0.528)	0.660
	Duration of consumption per month	0.960 (1.502–0.613)	0.857

* Statistically significant at *p* < 0.05.

** Statistically highly significant at *p* < 0.001.

## Leukoplakia group

Individuals who chewed unprocessed and processed forms of tobacco and areca nut were more likely (1.06 times) to experience leukoplakia than those who only chewed processed tobacco (OR = 1.08, 95% CI: 0.8–1.4). Individuals who chewed with a high frequency were more likely (four times) to experience leukoplakia than those with a low frequency (OR = 3.92, 95% CI: 2.78–5.92). Older individuals had a higher risk (1.7 times) than younger individuals to be affected by LP (OR = 1.71, CI: 1.3–2.2). Men were at 1.7 times more risk of getting LP than females (OR = 1.73, CI: 0.89–3.35). Those in the UL and L socio-economic classes were 1.5 times more at risk of getting LP than the upper classes (OR = 1.01–2.19). Individuals who chewed tobacco for a longer duration were more likely (1.37 times) to experience leukoplakia than those who chewed for a shorter duration (OR = 1.37, 95% CI: 0.96–1.96).

## OSMF group

Individuals who chewed tobacco and areca nut were more likely (1.9 times) to experience OSMF than those who only chewed tobacco (OR = 1.92, 95% CI: 1.52–2.51). Individuals who chewed with a high frequency were more likely (2.9 times) to experience OSMF than those with low frequency (OR = 2.9, 95% CI: 2.16–4.09). Individuals who chewed tobacco for a moderate duration were more likely (1.5 times) to experience OSMF than those who chewed for a shorter duration (OR = 1.54, 95% CI: 1.15–2.07). Older individuals had a 1.4 times higher risk of OSMF than younger individuals (OR = 1.4, CI: 1.12–1.77). Those in the UL and L socio-economic classes had a 1.1 times higher risk of getting OSMF than the upper classes (OR = 1.09, CI: 0.78–1.5).

## Quid-induced lichenoid reaction group

Individuals who chewed tobacco and areca nut were more likely (2.7 times) to experience QILR than those who only chewed tobacco (OR = 2.67, 95% CI: 1.69–4.2). Individuals who chewed unprocessed and processed forms of tobacco and areca nut were more likely (1.1 times) to experience QILR than those who only chewed processed tobacco (OR = 1.11, 95% CI: 0.64–1.9). Individuals who chewed tobacco with a high frequency were more likely (2.2 times) to experience QILR than those who chewed with a low frequency (OR = 2.18, 95% CI: 1.34–3.55). Older individuals had a 1.12 times higher risk of QILR than younger individuals (OR = 1.12, CI: 0.76–1.65). Men had 1.3 times more risk of getting QILR than women (OR = 1.28, CI: 0.74–2.21).

## Tobacco pouch keratosis group

Individuals who chewed tobacco with a high frequency were more likely (2.9 times) to experience TPK than those who chewed with a low frequency (OR = 2.9, 95% CI: 2.13–3.95). Older individuals had a 1.1 times higher risk of TPK than younger ones (OR = 1.1, CI: 0.9–1.36). Men had 2.9 times more risk of getting TPK than women (OR = 2.13, CI: 1.53–4.76). Individuals who chewed tobacco for a moderate duration were more likely (1.5 times) to experience TPK than those who chewed for a shorter duration (OR = 1.55, 95% CI: 1.17–2.06).

## Discussion

This study focuses on the epidemiology of quid usage and its correlation with OMLs among the population of western Rajasthan. The findings reveal significant associations between different types and forms of quid usage and the development of lesions such as LP, OSMF, QILR, and TPK. These results align with previous studies conducted in similar settings, providing a comprehensive understanding of the risk factors associated with quid-induced OMLs (21, 22).

The study showed that the most common age group affected by OMLs was 26–35 years, followed by 16–25 and 36–45 years. This pattern suggests that younger adults in western Rajasthan are particularly susceptible to OMLs due to quid use. The significant association between these age groups and the occurrence of lesions corroborates findings from other studies in India and Southeast Asia, where similar age groups were found to be at higher risk (23, 24). In India, Gupta et al. reported that OML prevalence was highest among individuals aged 20–40 years, reflecting a comparable demographic risk profile (5).

The higher prevalence among these age groups is often attributed to the early initiation of quid chewing in adolescence, leading to prolonged exposure by adulthood. Thomas et al. support the hypothesis that early initiation and longer duration of quid use significantly increase the risk of developing OMLs (25). The socio-economic pressures and lifestyle factors prevalent in this age group may also contribute to higher stress levels and the adoption of quid chewing as a coping mechanism (1).

This study found that men were significantly more affected by OMLs than women, consistent with existing literature on quid usage (24). This gender disparity is likely due to cultural norms in western Rajasthan, where quid chewing is more socially accepted among men. Warnakulasuriya et al. also found that men were more likely to use quid and develop related oral lesions compared to women (1). A study by Tsai et al. and Lee et al. similarly reported a higher prevalence of OMLs among male quid users, reinforcing the idea that men are at greater risk due to higher consumption rates and longer durations of use (10, 15).

The biological response to quid components may differ between genders, with men possibly exhibiting a greater propensity for developing lesions due to factors such as higher rates of smoking and alcohol consumption, common co-factors in male populations in South Asia (22). This gender-specific vulnerability suggests the need for tailored public health strategies to address the unique risks faced by male quid users.

SES emerged as a significant determinant of OML occurrence, with individuals from the UL and LM SES classes being more affected. This finding is consistent with previous research, such as the study by Boffetta et al., which highlighted that lower SES groups have higher rates of quid usage, partly due to the lower cost and greater accessibility of these products (7). In addition, these groups often have limited access to healthcare and education about the risks associated with quid use, leading to higher susceptibility to OMLs.

Similar patterns have been reported in studies conducted in Sri Lanka and Pakistan, where higher incidences of OMLs were observed in lower SES groups, supporting the findings of this study (3, 26).

These results underscore the need for public health initiatives focusing on reducing quid use in economically disadvantaged communities and improving access to healthcare and education.

This study highlights that the combination of tobacco and areca nut was the most common type of quid used and was significantly associated with the occurrence of lesions such as LP, OSMF, and TPK. This finding aligns with studies conducted in South Asia, where the use of areca nut with tobacco is wide spread and has been linked to a higher risk of developing OMLs. Nair et al. identified this combination as a major risk factor for OSMF, particularly in Indian populations (8).

The processed form of quid was the most frequently used, significantly associated with various lesions. This result is consistent with a study by Javed et al., which showed that processed quid products often contain higher concentrations of carcinogenic substances, leading to an increased risk of oral lesions (14). The additives and flavor enhancers used in processed quid products can enhance the absorption of carcinogens, as noted by Chung et al., who found a higher incidence of oral cancer in individuals who used processed quid (9).

The significant correlation between processed quid and the occurrence of OMLs suggests that the additives and chemical modifications involved in processing may heighten the risk of oral pathology.

Similar observations were made in Taiwan and Malaysia, where processed quid products were linked to higher rates of OMLs and oral cancer (2, 23). These findings underscore the need for stricter regulations on the production and sale of processed quid products.

High-frequency quid use (4–35 times per day) was significantly associated with the occurrence of OMLs, a finding supported by previous studies (6). Petersen et al. demonstrated that high-frequency chewing significantly increases the risk of OMLs due to repeated exposure to harmful substances (12).

The study also found that moderate to long durations of quid retention in the mouth were associated with a higher incidence of OMLs, corroborating the findings of Tsai et al., who showed that prolonged contact between quid and the oral mucosa increases the risk of lesion formation (10).

The correlation between long-term quid use and the development of OMLs was highlighted by Lee et al., who found that individuals with a history of more than 10 years of quid use were at significantly higher risk of developing oral cancer (2). These findings emphasize the importance of interventions aimed at reducing both the frequency and duration of quid use to lower the prevalence of OMLs in at-risk populations.

### Specific lesions and risk factors

### Leukoplakia

This study found that individuals chewing a combination of tobacco and areca nut in both unprocessed and processed forms are more likely to experience LP. This result aligns with findings from Warnakulasuriya et al., who reported a strong association between the combined use of tobacco and areca nut and the development of LP (1). In addition, this study found that older age groups were more susceptible to LP, consistent with Shah et al., who observed that the risk of LP increases with age due to prolonged exposure to carcinogens (3).

Men were found to be at higher risk of developing LP, reflecting similar gender-based findings in studies by Lee et al. and Chiu et al., both of which reported a higher prevalence of LP among male quid users (2, 6). The increased risk of LP in individuals from the UL and LM SES classes further supports the findings of earlier studies that highlighted socio-economic disparities in oral health outcomes (5).

### Oral submucous fibrosis

OSMF was more likely to occur in individuals who chewed combination of tobacco and areca nut, particularly in their processed forms. This finding is consistent with the results of studies by Nair et al. and Murti et al., which identified this combination as a major risk factor for OSMF (8, 11). High-frequency chewing and long durations of use were also significantly associated with OSMF, supporting the findings of Thomas et al., who demonstrated that chronic exposure to these substances leads to progressive fibrosis of the oral mucosa (16).

This study's finding that older age groups were at higher risk of OSMF aligns with similar observations in studies by Shah et al. and Gupta et al., where older individuals were more susceptible to OSMF due to longer exposure times (3, 5). The slight increase in risk among individuals from the UL and LM SES classes highlights the socio-economic dimensions of OSMF, as reported by Boffetta et al. and Jacob et al. (7, 13).

### Quid-induced lichenoid reaction

The association between QILR and the combined use of tobacco and areca nut was significant, consistent with findings from earlier studies. Winstock et al. found that QILR was more prevalent among individuals who used both tobacco and areca nut compared to those who used tobacco alone (22). The inflammatory nature of QILR suggests that the irritant properties of these substances, especially in their processed forms, may trigger immune-mediated responses, leading to lesion formation.

High-frequency quid use was significantly associated with QILR, supporting the findings of Lee et al., who reported a higher incidence of QILR among individuals who chewed quid multiple times per day (2). This study also found that men and older individuals were slightly more at risk, although these associations were less pronounced than those observed for other lesions. This result is consistent with research by Thomas et al., who found a similar pattern of QILR prevalence among male quid users (25).

### Tobacco pouch keratosis

TPK was more likely to occur in individuals who chewed tobacco frequently, particularly in processed forms. This finding aligns with the results of studies by Chung et al. and Javed et al., which identified high-frequency chewing and the use of processed tobacco products as significant risk factors for TPK (9, 14). The association between TPK and longer durations of quid retention in the mouth further supports the findings of Warnakulasuriya et al., who showed that prolonged contact between tobacco and the oral mucosa increases the risk of keratotic lesions (1).

This study's finding that men were more likely to develop TPK than women is consistent with the results of studies by Shah et al. and Petersen et al., both of which reported a higher prevalence of TPK among male quid users (12, 19). The association between TPK and socio-economic status, with a higher risk observed for individuals from the UL and LM classes highlights the importance of considering socio-economic factors in the prevention and treatment of TPK, as suggested by earlier research.

This study has some limitations. The study was cross-sectional in nature and could, at best, provide a glimpse of the situation. The survey was conducted among patients who visited a dental college in western Rajasthan and hence the findings should be interpreted with caution.

## Conclusion

The findings of this study contribute to a deeper understanding of the complex interplay between quid usage patterns and the development of oral mucosal lesions. By comparing these results with the existing literature, it is evident that the combination of tobacco and areca nut, especially in processed forms, poses a significant risk for the development of lesions such as leukoplakia, oral sub mucous fibrosis, quid-induced lichenoid reactions, and tobacco pouch keratosis. The significant associations with factors such as age, gender, socio-economic status, frequency of use, and duration of quid retention in the mouth underscore the need for targeted public health interventions that address these specific risk factors. Continued research and collaboration across regions will be essential in developing effective strategies to reduce the burden of quid-induced oral health issues.

## Data Availability

The original contributions presented in the study are included in the article/Supplementary Material, further inquiries can be directed to the corresponding author.
